# Adrenal Vein Sampling for Conn’s Syndrome: Diagnosis and Clinical Outcomes

**DOI:** 10.3390/diagnostics5020254

**Published:** 2015-06-19

**Authors:** Amy R. Deipolyi, Alexander Bailin, Stephan Wicky, Shehab Alansari, Rahmi Oklu

**Affiliations:** 1 Division of Vascular & Interventional Radiology, Department of Radiology, New York University Medical Center, New York, NY 10016, USA; E-Mail: amy.deipolyi@nyumc.org; 2 Massachusetts General Hospital, Harvard Medical School, Division of Interventional Radiology, 55 Fruit Street, GRB-290A, Boston, MA 02114, USA; E-Mails: bhartealex@gmail.com (A.B.); stephan.wickyvandoyer@umassmemorial.org (S.W.); drshehabalansari@gmail.com (S.A.)

**Keywords:** adrenal vein sampling, primary aldosteronism, Conn’s syndrome, endocrinology

## Abstract

Adrenal vein sampling (AVS) is the gold standard test to determine unilateral causes of primary aldosteronism (PA). We have retrospectively characterized our experience with AVS including concordance of AVS results and imaging, and describe the approach for the PA patient in whom bilateral AVS is unsuccessful. We reviewed the medical records of 85 patients with PA and compared patients who were treated medically and surgically on pre-procedure presentation and post-treatment outcomes, and evaluated how technically unsuccessful AVS results were used in further patient management. Out of the 92 AVS performed in 85 patients, AVS was technically successful bilaterally in 58 (63%) of cases. Either unsuccessful AVS prompted a repeat AVS, or results from the contralateral side and from CT imaging were used to guide further therapy. Patients who were managed surgically with adrenalectomy had higher initial blood pressure and lower potassium levels compared with patients who were managed medically. Adrenalectomy results in significantly decreased blood pressure and normalization of potassium levels. AVS can identify surgically curable causes of PA, but can be technically challenging. When one adrenal vein fails to be cannulated, results from the contralateral vein can be useful in conjunction with imaging and clinical findings to suggest further management.

## 1. Introduction

Primary aldosteronism (PA) is a potentially curable cause of hypertension in 5%–14.4% of the hypertensive population [1,2,3]. In PA, excess aldosterone acts on renal aldosterone receptors, causing reabsorption of salt and water and excretion of potassium. This causes refractory hypertension and potentially hypokalemic metabolic acidosis. Cardiomyocytes, cardiac fibroblasts, and vascular smooth muscle cells also contain aldosterone receptors [4]. Chronically elevated aldosterone levels are associated with an increased risk of cardiac events [5]. There are at least seven subtypes of PA. Aldosterone-producing adenomas (APA) and bilateral adrenal hyperplasia (BAH, also referred to as “idiopathic aldosteronism”) account for 98% of cases [6]. The remaining 2% include rare familial subtypes, unilateral primary adrenal hyperplasia (PHA), and adrenal carcinoma. Management depends on differentiating these subtypes; unilateral etiologies may be cured surgically, whereas bilateral causes are managed medically with antihypertensives, including mineralocorticoid receptor antagonists. Patients with confirmed PA are evaluated with cross sectional imaging and biochemical assays. CT and MRI do not reliably distinguish unilateral from bilateral disease [7,8,9,10,11,12] but can help guide management in difficult cases and identify large adrenal masses concerning for malignancy. Prediction models have been developed to predict which patients have unilateral APAs, though have failed to distinguish such cases in the elderly [13]. Adrenal vein sampling (AVS), on the other hand, is the gold standard for localizing autonomous sources of aldosterone.

AVS is technically challenging with high reported failure rates, usually due to difficulty cannulating the right adrenal vein, which originates from the inferior vena cava [14,15]. When the adrenal vein is successfully cannulated, elevated cortisol levels are present in the adrenal vein sample. The cortisol level in the adrenal vein (C_AV_) is compared to peripheral samples, for example from the IVC (C_IVC_), to generate a ratio (C_AV_:C_IVC_). There is considerable lack of standardization in interpreting results, with a wide range of C_AV_:C_IVC_ index cutoffs from 1.1 to 5 [16]. There is also controversy regarding the threshold determining unilateral from bilateral hypersecretion [12,15,17,18,19]. Most advocate for comparing the cortisol-corrected aldosterone output from each gland [12,15,19] whereas others advocate for identifying contralateral gland suppression [17,18]. There is also debate regarding the use of ACTH stimulation, omitted by some and advocated by others stressing the importance of ACTH to minimize aldosterone production fluctuations induced by stress [20]. In light of the difficulty in reliably cannulating both adrenal veins, some have considered using limited AVS data to identify surgical candidates [17,18].

This study retrospectively characterizes our single institution experience with AVS including concordance of AVS results with imaging and approaching a PA patient in whom bilateral AVS is unsuccessful.

## 2. Materials and Methods

### 2.1. Subjects

In this IRB-approved, HIPAA-compliant retrospective study, patients who underwent AVS between March 1990 and May 2014 were identified using the radiology department’s electronic searchable database using the keyword “adrenal vein sampling”. All patients were previously diagnosed with PA by specialized endocrinologists, according to accepted diagnostic standards [10]. Medical records were reviewed for demographics, blood pressure (BP, in mmHg), number of antihypertensive medications, potassium level (mEq/L), adrenal cross sectional imaging, AVS details and results, adrenalectomy, and pathologic findings. Pre-treatment BP and number of antihypertensive medications were assessed on initial presentation for medically managed patients or prior to adrenalectomy for surgically managed patients. Post-treatment BP and number of antihypertensive medications were assessed at the 1-month post-AVS clinic visit for medically managed patients, or at the 1-month post-adrenalectomy clinic visit for surgically treated patients.

### 2.2. Procedure

There were a total of 13 operators who performed AVS over the study period. AVS was performed in a sequential stimulated manner [12]. Briefly, medications including mineralocorticoid receptor antagonists were administered at least 2 weeks before AVS. The common femoral vein was catheterized for access to the adrenal veins, which were selected sequentially. Samples were taken separately from each adrenal vein and from the infrarenal inferior vena cava (IVC) or common femoral vein to measure cortisol (C) and aldosterone (A). Sequential adrenal vein sampling was performed at least 30 min after initiation of a continuous infusion of Cosyntropin (Mylan Institutional, Rockford, IL, USA), synthetic ACTH.

Technically successful cannulation of the adrenal vein was determined by a cortisol ratio criteria of C_AV_:C_IVC_ > 3. A normalized aldosterone (A/C) in the dominant adrenal vein divided by the nondominant adrenal gland (lateralization index) of 4 or more was used to define the presence of APA or PHA. In the case of unsuccessful bilateral AVS, the decision to proceed with repeat AVS, surgery or medical management was based on correlation of medical imaging with unilateral AVS data, by the referring physician.

### 2.3. Imaging

Radiology reports from the most recent CT or MRI prior to AVS were evaluated. Imaging reports were categorized in one of four ways: left-sided lesion, right-sided lesion, bilateral lesions or nodularity, or no lesions. CT findings were compared with AVS findings, and ultimately with pathologic findings.

### 2.4. Outcomes and Data Analysis

Pathology records were reviewed for the patients who underwent surgery. Pathology was considered concordant when an adenoma was discovered on the side of a CT finding, or lateralization suggested by AVS. Patient outcome was analyzed on the basis of both decrease in BP and change in antihypertensive drug prescriptions, assessed at the time of the 1-month post-procedure or post-surgical follow up visit. For both surgically treated and medically managed groups, the pre- and post-systolic BP (SBP), diastolic BP (DBP) and number of antihypertensive medications were compared with the Wilcoxon matched-pairs signed rank test within groups. Between groups, the post-treatment SBP and DBP were compared with unpaired *t* tests, χ^2^ tests, and the numbers of post treatment antihypertensive medications were compared with a Mann Whitney U test. All statistical tests were performed with Prism (Version 4.0, GraphPad Software Inc, San Diego, CA, USA). For all tests, *p* < 0.05 was considered statistically significant.

## 3. Results

### 3.1. Patient Demographics

**Figure 1:**
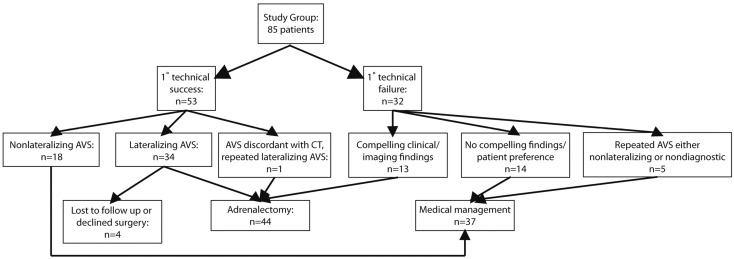
Flow diagram illustrating study patients. There were 85 patients included in the study. Workup and outcomes are presented.

A total of 85 patients (37 F, 48 M) with a mean age of 50.6 (range 29–76) years underwent 92 AVS procedures (Figure 1). In 5 patients, AVS was repeated once and in one patient AVS was repeated twice. All patients were hypertensive on initial presentation prior to AVS, with overall initial mean blood pressure of 160/93 (SE: 3.2/1.7).

A total of 44 patients were managed surgically with adrenalectomy. Four patients (2 men, 2 women) had technically successful, lateralizing AVS, but were lost to follow up. The remaining 37 patients were managed medically. Demographic and laboratory assessment of these patients prior to AVS are presented in Table 1. Patients who were eventually managed surgically had significantly higher blood pressure according to unpaired *t* tests and were more likely to be hypokalemic according to χ^2^ tests than those managed medically. In both groups, patients were on a similar initial number of antihypertensive medications (3, range 0–6).

**Table 1:** Pre-AVS demographic and clinical data. Patients who eventually underwent surgery had significantly higher initial blood pressure (mmHg) and were more likely to be hypokalemic.

Demographic and Clinical Variable	Surgical	Medical	*p* value
Mean Age (SE)	51.2 (1.5)	50 (1.6)	0.7
% Male	45%	70%	0.04
Pre-AVS Systolic BP (SE)	167 (4)	150 (4)	0.009
Pre-AVS Diastolic BP (SE)	96 (2)	89 (3)	0.03
Pre-AVS BP meds (range)	3 (1–6)	2 (0–5)	0.02
% Hypokalemic	86%	54%	0.003
Pre-AVS Potassium (SE)	3.3 (0.1)	3.6 (0.1)	0.006
Pre-AVS Aldosterone:Renin Ratio (SE)	69 (14)	94 (19)	0.3

SE: Standard error of the mean; AVS: Adrenal vein sampling; BP: Blood pressure.

### 3.2. AVS Findings and Concordance to Imaging and Pathology

Using the C_AV_:C_IVC_ > 3 threshold as technically successful AVS procedures, only 58 of 92 (63%) procedures met this criteria. Of the 34 (37%) technical failures, the majority were unsuccessful cannulations involving the right adrenal vein only (82%); the remainder involved the left adrenal vein only (6%) or both adrenal veins (12%). Over time, an increasing number of AVS procedures have been performed at our institution. Among the 13 operators who performed AVS, there has been wide variation in success rates; average technical success was 82% (SE 8%, range 0%–100%) between operators. Recently, however, rotational angiography (DynaCT Artis Zeego; Siemens, Malvern, PA, USA) was incorporated into practice; since 10/2012, there has been 100% technical success.

In total, 44 patients (44 AVS procedures) were eventually surgically managed, 37 patients were medically managed (43 AVS procedures), and 4 patients (4 procedures) were lost to follow up (Table 2). Among the 44 surgical patients, there were 32 technically successful AVS procedures. All of the 31 procedures lateralized to one side (17 left and 14 right). Therefore, there were a total of 31 procedures with subsequent pathologic evaluation among 31 patients. In one patient, CT showed a 1.5-cm left adrenal mass with enhancement that persisted on 10-min delays, but initial AVS was suggestive of a right-sided lesion. Given the contradictory findings, AVS was repeated and diagnosed a left-sided lesion; thus the patient went on to have a left adrenalectomy. Pathology showed a left cortical adenoma; BP decreased from 164/98 to 118/78 and the patient’s hypokalemia resolved, with no change in the number of antihypertensives (1).

Overall, 29 of 31 patients with diagnostic lateralizing AVS procedures had pathologically diagnosed adrenocortical adenomas, giving an accuracy of 94%. Of these, 4 were described as an adrenocortical adenoma with a background of nodular hyperplasia. The remaining 2 patients had multinodular adrenal hyperplasia, without a dominant nodule described. Among the 31 patients with diagnostic lateralizing AVS, 3 had CTs demonstrating no lesion and 1 had a CT demonstrating bilateral lesions; thus AVS provided essential diagnostic information beyond cross-sectional imaging in these 4 patients.

CT imaging was performed in all 44 surgically treated patients; one patient did not have documented surgical pathology, giving 43 patients with CT imaging and pathologic follow up. In 3 of these patients, no adrenal abnormalities were detected on cross-sectional imaging despite adenomas found at surgery, giving a false negative rate of 7%. Of the 21 patients with diagnostic nonlateralizing AVS, 11 had unilateral lesions demonstrated on CT imaging. Thus in 14 of 63 patients (22%) with diagnostic AVS, AVS resulted in management. 

Thirteen patients underwent adrenalectomy despite technically unsuccessful AVS. One patient’s pathology was not available, giving a total of 12 patients who underwent 12 AVS with subsequent surgical resection. In all of these patients, the right adrenal vein was not adequately sampled based on cortisol ratio criteria, *i.e.*, C_AV_:C_IVC_ < 3, with average selectivity indices of 1.3 (SE 0.1, range 0.8–2.6). In one patient, large respiratory tidal volume led to significant motion causing limited cannulation time of the right adrenal vein for sampling. Surgery was performed based on cross-sectional imaging in these patients, in conjunction with compelling clinical and laboratory findings. Overall, for the 12 patients with technically unsuccessful right adrenal vein cannulations, CT was concordant with pathology in 11 of 12 cases (92%). Among patients with technically unsuccessful right adrenal vein cannulation and left-sided adenomas found on imaging and at surgery, the left adrenal vein aldosterone:cortisol ratio was on average 22.6 (SE 8.9, range 2.1–58.4). In contrast, among patients with technically unsuccessful right adrenal cannulation and right-sided adenomas found on imaging and at surgery, the left adrenal vein aldosterone:cortisol ratio was on average 4.5 (SE 4.0, range 0.3–24.4). Therefore, high left adrenal venous aldosterone:cortisol ratios in conjunction with corroborating imaging evidence may help suggest a left-sided APA in challenging cases, where lower ratios would suggest a right-sided lesion.

Among the 37 patients who were managed medically, there were 43 AVS procedures. Four patients had 2 AVS procedures and one patient had 3 procedures, due to technical failures on first attempts. Two of these patients with 2 procedures and the one patient with 3 procedures eventually had successful bilateral AVS, while the other 2 patients had 2 technically unsuccessful procedures. In total, there were 21 technically unsuccessful AVS cases among the medically managed patients. Of these, 16 involved the right adrenal vein only, 2 involved the left adrenal vein only, and 3 were bilateral failures. In two of the patients with repeated studies, initial AVS lateralized to the left adrenal vein, though no adenoma had been detected on CT. They both went on to have further AVS showing no lateralization, prompting medical therapy. In 18 patients, AVS was technically successful and non-lateralizing, and thus were medically managed, despite 5 of these patients having suggestive CT findings. In the 14 patients with unsuccessful AVS who did not undergo repeat sampling, the absence of contralateral adrenal suppression was taken as evidence for absence of operable lesions.

**Table 2:** AVS, pathologic, imaging and clinical data for all patients. SI: sensitivity index; LI: lateralization index; SBP: systolic blood pressure; DBP: diastolic blood pressure; HypoK: hypokalemia; Preop: pre-adrenalectomy; Postop: post-adrenalectomy; meds: blood pressure medications. Hypokalemia was considered resolved in potassium levels increased from a value below normal to a value within normal range or higher (normal range 3.5–5.0 mEq/L). *** indicates patients who underwent repeated AVS.

**CT Findings**	**SI (L)**	**SI (R)**	**LI (L)**	**LI (R)**	**SBP pre**	**DBP pre**	**SBP Post**	**DBP Post**	**Preop Meds**	**Postop Meds**	**HypoK Resolved**	**Adrenal Resected**	**Pathology**
**Lateralizing AVS, Adrenalectomy (*n* = 31)**													
Left adrenal adenoma	**9.5**	**51.3**	**127.8**	**0.01**	204	102	-	-	2	-	yes	Left	Adrenocortical adenoma
Left adrenal adenoma	**32.7**	**30.8**	**58.0**	**0.02**	170	90	150	92	4	0	yes	Left	Adrenocortical adenoma
Left adrenal lesion too small to characterize	**108.5**	**100.2**	**54.1**	**0.02**	174	108	120	70	4	3	yes	Left	Adrenocortical adenoma
Normal	**19.3**	**48.8**	**28.3**	**0.04**	144	90	118	68	4	1	yes	Left	Adrenocortical adenoma
Left adrenal adenoma	**52.6**	**70.8**	**27.2**	**0.04**	130	80	107	73	6	0	yes	Left	Adrenocortical adenoma
Normal	**38.9**	**54.9**	**26.0**	**0.04**	130	80	118	78	2	0	yes	Left	Adrenocortical adenoma
Left adrenal adenoma	**12.0**	**19.7**	**22.9**	**0.04**	170	98	140	60	2	1	yes	Left	Adrenocortical adenoma
Left adrenal adenoma	**23.3**	**31.1**	**22.6**	**0.04**	154	96	102	78	5	1	yes	Left	Adrenocortical adenoma
Left adrenal adenoma	**18.6**	**48.7**	**13.6**	**0.1**	158	108	120	70	2	0	yes	Left	Adrenocortical adenoma
Left adrenal	**44.7**	**50.8**	**13.6**	**0.1**	164	98	109	67	1	1	yes	Left	Adrenocortical adenoma
adenoma ***													
Left adrenal adenoma	**50.6**	**39.8**	**10.1**	**0.1**	155	90	110	60	2	0	yes	Left	Adrenocortical adenoma
Left adrenal adenoma	**5.8**	**5.5**	**9.4**	**0.1**	130	85	130	85	4	2	yes	Left	Adrenocortical adenoma
Left adrenal adenoma	**30.5**	**8.0**	**5.0**	**0.2**	200	115	134	90	2	1	yes	Left	Adrenocortical adenoma
Left adrenal adenoma	**35.4**	**22.1**	**3.9**	**0.3**	200	100	120	70	2	1	yes	Left	Adrenocortical adenoma
Left adrenal thickening	**6.1**	**10.6**	**33.0**	**0.0**	-	-	-	-	5	2	yes	Left	Adrenal cortical hyperplasia with mixed macro- and micronodular pattern
Left adrenal adenoma	**49.2**	**39.1**	**11.8**	**0.1**	160	88	117	76	3	0	yes	Left	Adrenocortical adenoma with a background of nodular cortical hyperplasia
**CT Findings**	**SI (L)**	**SI (R)**	**LI (L)**	**LI (R)**	**SBP pre**	**DBP pre**	**SBP Post**	**DBP Post**	**Preop meds**	**Postop meds**	**HypoK Resolved**	**Adrenal Resected**	**Pathology**
Left adrenal lesion too small to characterize	**18.5**	**35.2**	**12.6**	**0.1**	150	100	102	72	3	2	yes	Left	Adrenocortical adenoma with a background of nodular cortical hyperplasia
Right adrenal adenoma	**9.4**	**21.9**	**0.04**	**23.6**	188	90	140	100	3	3	yes	Right	Adrenocortical adenoma
Right adrenal adenoma	**16.2**	**18.8**	**0.2**	**4.5**	168	88	140	100	3		yes	Right	Adrenocortical adenoma
Right adrenal adenoma	**42.0**	**65.6**	**0.2**	**4.7**	166	90	138	68	5	2	yes	Right	Adrenocortical adenoma
Right adrenal adenoma	**9.3**	**38.7**	**0.2**	**5.1**	160	98	148	88	2	1	yes	Right	Adrenocortical adenoma
Right adrenal adenoma	**22.1**	**39.2**	**0.1**	**10.3**	200	110	130	82	4	2	yes	Right	Adrenocortical adenoma
Normal	**39.3**	**31.7**	**0.1**	**11.6**	180	110	118	80	2	2	yes	Right	Adrenocortical adenoma
Right adrenal adenoma	**17.5**	**11.7**	**0.1**	**14.3**	150	90	130	80	5	4	yes	Right	Adrenocortical adenoma
Right adrenal adenoma	**85.6**	**49.7**	**0.1**	**14.7**	-	-	-	-	2		yes	Right	Adrenocortical adenoma
Right adrenal adenoma	**16.5**	**39.7**	**0.1**	**17.9**	226	141	140	84	2	0	yes	Right	Adrenocortical adenoma
Right adrenal adenoma	**8.5**	**23.3**	**0.03**	**36.3**	162	85	122	76	4	1	yes	Right	Adrenocortical adenoma
Right adrenal adenoma	**50.3**	**9.1**	**0.1**	**16.5**	170	90	134	80	1	1	yes	Right	Adrenocortical adenoma with a background of nodular cortical hyperplasia
Right adrenal adenoma	**11.7**	**25.6**	**0.04**	**22.7**	140	90	117	83	1	1	yes	Right	Adrenocortical adenoma with a background of nodular cortical hyperplasia
Right adrenal adenoma	**10.7**	**10.7**	**0.1**	**14.1**	190	110	120	90	3	1	yes	Right	Adrenocortical adenoma with a background of nodular cortical hyperplasia
Bilateral adrenal thickening	**16.9**	**10.8**	**0.03**	**31.7**	160	90	-	-	2	1	yes	Right	Multinodular adrenal cortical hyperplasia
**CT Findings**	**SI (L)**	**SI (R)**	**LI (L)**	**LI (R)**	**SBP pre**	**DBP pre**	**SBP Post**	**DBP Post**	**Preop meds**	**Postop meds**	**HypoK Resolved**	**Adrenal Resected**	**Pathology**
**Nondiagnostic AVS, Adrenalectomy (*n* = 13)**													
Bilateral adrenal adenomas	**4.8**	**1.2**	**2.9**	**0.3**	-	-	136	90	3	1	yes	Bilateral (subtotal right)	Bilateral nodular cortical hyperplasia
Left adrenal adenoma	**13.6**	**0.8**	**34.9**	**0.0**	150	90	119	77	3	1	yes	Left	Adrenocortical adenoma
Left adrenal adenoma	**17.2**	**0.9**	**31.4**	**0.0**	142	71	123	68	4	2	yes	Left	Adrenocortical adenoma
Bilateral adrenal adenomas	**7.6**	**1.2**	**6.1**	**0.2**	-	-	121	66	3	0	yes	Left	Adrenocortical adenoma
Left adrenal adenoma	**33.1**	**2.6**	**12.4**	**0.1**	177	117	100	70	3	0	yes	Left	Cortical nodular hyperplasia with two dominant nodules
Normal	**2.3**	**1.0**	**4.4**	**0.2**	-	-	115	66	-	4	no	Left	Not documented
Left adrenal adenoma	**7.3**	**1.5**	**2.1**	**0.5**	148	96	130	80	2	1	yes	Left	Nodular hyperplasia with a dominant adrenocortical adenoma
Bilateral adrenal adenomas	**3.1**	**1.5**	**48.9**	**0.0**	150	87	124	76	3	3	yes	Right	Adrenal cortical hyperplasia with a dominant nodule
Right adrenal adenoma	**17.4**	**1.0**	**1.4**	**0.7**	120	73	116	70	3	2	-	Right	Adrenocortical adenoma
Right adrenal adenoma	**7.1**	**1.5**	**0.5**	**2.1**	154	92	155	105	2	0	yes	Right	Adrenocortical adenoma
Right adrenal adenoma	**43.9**	**1.0**	**0.3**	**3.7**	240	120	122	86	2	1	yes	Right	Adrenocortical adenoma
Right adrenal adenoma	**23.6**	**1.2**	**0.1**	**12.8**	240	120	138	68	5	2	yes	Right	Adrenocortical adenoma
Right adrenal adenoma	**16.8**	**1.8**	**0.0**	**47.7**	133	77	124	78	3	0	no	Right	Adrenocortical adenoma
**CT Findings**	**SI (L)**	**SI (R)**	**LI (L)**	**LI (R)**	**SBP pre**	**DBP pre**	**SBP post**	**DBP post**	**PreAVS meds**	**PostAVS meds**	**-**		
**Nonlateralizing Diagnostic AVS, Medically Managed (*n* = 21)**													
Normal	**27.3**	**41.9**	**0.3**	**3.6**	110	78	128	90	4	6	-		
Normal	**39.5**	**76.2**	**0.5**	**2.1**	163	108	120	90	3	3	-		
Normal	**48.7**	**33.7**	**0.5**	**2.0**	154	100	118	88	0	2	-		
Normal	**84.3**	**92.9**	**0.5**	**1.9**	158	80	116	64	2	3	-		
Normal	**24.7**	**101.9**	**0.5**	**1.8**	144	100	154	89	2	4	-		
Normal	**35.1**	**43.0**	**1.6**	**0.6**	130	72	150	95	1	3	-		
Normal	**30.1**	**22.9**	**3.5**	**0.3**	140	100	140	105	1	2	-		
Bilateral adrenal adenomas	**78.2**	**53.6**	**0.5**	**2.0**	146	70	124	76	3	5	-		
Bilateral adrenal adenomas	**70.1**	**87.7**	**0.9**	**1.1**	155	96	132	77	3	4	-		
Bilateral adrenal thickening	**11.9**	**22.0**	**0.8**	**1.3**	150	80	148	75	1	4	-		
Right adrenal adenoma	**23.5**	**27.9**	**0.4**	**2.3**	150	97	128	71	3	4	-		
Right adrenal adenoma	**7.0**	**7.0**	**1.0**	**1.0**	150	74	140	74	3	6	-		
Right adrenal adenoma	**23.1**	**35.5**	**1.0**	**1.0**	240	120	100	80	1	3	-		
Right adrenal thickening	**52.3**	**59.2**	**0.4**	**2.6**	155	88	140	70	1	4	-		
Left adrenal adenoma	**37.7**	**46.4**	**0.3**	**3.4**	160	90			2		-		
Left adrenal adenoma	**75.2**	**74.4**	**2.0**	**0.5**	137	86	134	82	3	4	-		
Left adrenal adenoma	**23.4**	**38.0**	**3.2**	**0.3**	140	86	158	100	4	4	-		
Left adrenal adenoma and right adrenal thickening	**40.2**	**39.5**	**0.3**	**3.3**	148	102	136	84	2	7	-		
**CT Findings**	**SI (L)**	**SI (R)**	**LI (L)**	**LI (R)**	**SBPpre**	**DBPpre**	**SBPpost**	**DBPpost**	**PreAVS meds**	**PostAVS meds**	**-**		
Left adrenal	**23.7**	**1.1**	**2.0**	**0.5**	170	90	125	74	2	4	-		
adenoma ***													
Left adrenal	**8.6**	**0.9**	**0.6**	**1.6**	171	117	142	91	3	4	-		
thickening ***													
Left adrenal	**1.4**	**35.1**	**6.2**	**0.2**	158	94	140	76	2	4	-		
adenoma ***													
**CT Findings**	**SI (L)**	**SI (R)**	**LI (L)**	**LI (R)**	**SBPpre**	**DBPpre**	**SBPpost**	**DBPpost**	**PreAVS meds**	**PostAVS meds**	**Rationale for Medical Management**		
**Lateralizing AVS, no Follow Up or Medically Managed (*n* = 4)**													
Not documented	**28.2**	**32.0**	**9.4**	**0.1**	154	88	-	-	0	-	Lost to follow up		
Not documented	**47.1**	**50.7**	**37.2**	**0.0**	-	-	-	-	-	-	Lost to follow up		
Bilateral adrenal adenomas	**15.0**	**27.3**	**0.2**	**4.1**	152	90	180	72	3	3	Patient deferring surgery		
Not documented	**17.8**	**60.9**	**10.2**	**0.1**	-	-	-	112	68	1	Patient deferring surgery		
**Nondiagnostic AVS, Medically Managed (*n* = 16)**													
Bilateral adrenal thickening	**21.4**	**0.9**	**0.2**	**6.1**	201	124	140	90	0	3	Clinical/imaging data not compelling, AVS not repeated		
Left adrenal adenoma	**87.6**	**1.5**	**0.2**	**6.4**	158	103	130	71	-	-	Clinical/imaging data not compelling, AVS not repeated		
Left adrenal thickening	**31.8**	**0.9**	**1.0**	**1.0**	134	70	150	88	2	4	Clinical/imaging data not compelling, AVS not repeated		
Left adrenal thickening	**1.5**	**11.2**	**5.2**	**0.2**	118	83	110	78	2	2	Clinical/imaging data not compelling, AVS not repeated		
Normal	**1.4**	**2.3**	**2.8**	**0.4**	120	72	106	70	2	3	Clinical/imaging data not compelling, AVS not repeated		
CT Findings	**SI (L)**	**SI (R)**	**LI (L)**	**LI (R)**	SBPpre	DBPpre	SBPpost	DBPpost	PreAVS meds	PostAVS meds	Rationale for Medical Management		
**Lateralizing AVS, no Follow Up or Medically Managed (*n* = 4)**													
Normal	**35.4**	**0.9**	**2.6**	**0.4**	144	96	135	85	2	2	Clinical/imaging data not compelling, AVS not repeated		
Normal	**27.2**	**1.5**	**1.1**	**0.9**	140	80	134	70	2	3	Clinical/imaging data not compelling, AVS not repeated		
Normal	**53.4**	**1.0**	**1.3**	**0.7**	120	80	140	78	4	4	Clinical/imaging data not compelling, AVS not repeated		
Right adrenal adenoma	**10.0**	**0.9**	**2.9**	**0.3**	100	70	114	70	2	3	Clinical/imaging data not compelling, AVS not repeated		
Right adrenal adenoma	**25.6**	**1.5**	**0.6**	**1.5**	160	70	133	74	5	6	Clinical/imaging data not compelling, AVS not repeated		
Right adrenal adenoma	**2.5**	**2.5**	**0.1**	**7.7**	150	80	131	62	5	6	Clinical/imaging data not compelling, AVS not repeated		
Right adrenal thickening	**42.0**	**1.4**	**0.3**	**3.3**	140	96	134	84	2	3	Clinical/imaging data not compelling, AVS not repeated		
Right adrenal thickening	**1.0**	**1.0**	**0.9**	**1.1**	144	64	-	-	5	-	Clinical/imaging data not compelling, AVS not repeated		
Right adrenal adenoma	**49.2**	**1.3**	**0.1**	**10.3**	210	114	165	95	2	3	Refused repeat AVS or further workup		
Right adrenal adenoma ***	**15.8**	**2.5**	**3.3**	**0.3**	136	80	129	78	2	1	Repeated AVS also failed; no compelling imaging/clinical		
Normal ***	**35.0**	**2.4**	**0.2**	**5.9**	160	80	-	-	3	2	Failed AVS twice, refused further AVS		

### 3.3. Response to Therapy

Surgically managed patients experienced significantly improved potassium levels and blood pressure after adrenalectomy (Table 3). Mean potassium level increased from 3.3 (SE 0.1) mEq/L prior to the procedure to 4.1 (SE 0.1) mEq/L after adrenalectomy, which was significant according to a paired t test (*p* < 0.0001). SBP significantly decreased from 167 (SE 5) mmHg to 124 (SE 2) mmHg according to a paired *t* test (*p* < 0.0001), as did DBP from 97 (SE 2) mmHg to 78 (SE 2) mmHg (*p* < 0.0001). Finally, the mean number of antihypertensive medications decreased from 3 (range 1–6) drugs to 1 (range 0–4) drug, which was significantly different according to a Mann-Whitney test (*p* < 0.0001). At least 11 of 35 patients (31%) with documented post-surgical medication lists required no antihypertensive medications at all. Among patients who had adrenalectomy performed on the side indicated by AVS lateralization, all patients had a 10% or greater reduction in SBP; average decrease was 25% (SE 2%, range 10%–47%). All patients had resolution of hypokalemia after adrenalectomy. Post-operative follow up was on average 3.4 years (SE 10 months, range 1 month–17 years).

**Table 3:** Clinical and laboratory findings in patients before and after adrenalectomy. After surgery, blood pressure decreased on fewer antihypertensive medications, and potassium normalized. Potassium presented as mEq/L; BP as mmHg.

Outcome Measure	Before Surgery	After Surgery	*p* value
Potassium (SE)	3.3 (0.1)	4.1 (0.1)	*p* < 0.0001
Systolic BP (SE)	167 (5)	124 (2)	*p* < 0.0001
Diastolic BP (SE)	97 (2)	79 (2)	*p* < 0.0001
# BP meds (range)	3 (1–6)	1 (0–4)	*p* < 0.0001

SE: Standard error of the mean; BP: Blood Pressure.

Subset analysis of the 13 patients with technically unsuccessful AVS who nonetheless underwent adrenalectomy showed that SBP significantly decreased from 174 (SE 15) mmHg to 124 (SE 3) mmHg (*p* = 0.01) and DBP decreased from 102 (SE 5) mmHg to 79 (SE 3) mmHg (*p* = 0.01). The number of antihypertensive medications also decreased from 3 to 1 (*p* = 0.002). Potassium level significantly increased from 3.2 (SE 0.1) mEq/L to 4.1 (SE 0.2) mEq/L (*p* = 0.0002).

Medically managed patients also had improved blood pressure, with systolic pressure decreasing from 152 (SE 4) mmHg to 133 (3) mmHg after AVS, which was significantly different by paired *t* test (*p* = 0.002). Similarly, diastolic blood pressure also decreased from 84 (4) mmHg to 77 (3) mmHg (*p* = 0.001). The mean number of antihypertensives increased from 3 (range 0–6) to 5 (range 1–7) (*p* = 0.0007).

We also performed a subset analysis, including only patients with diagnostic AVS, who were either surgically (*n* = 31) managed after lateralizing study or medically managed (*n* = 21) after nonlateralizing study. While there was no difference between surgically and medically managed patients in SBP (167 *vs.* 154 mmHg; *p* = 0.05 by *t* test), DBP (97 *vs.* 92 mmHg, *p* = 0.2 by *t* test), or number of pre-AVS blood pressure medications (3 *vs.* 2, *p* = 0.07 by Mann Whitney *U* test), there was lower SBP (125 *vs.* 134 mmHg, *p* = 0.04 by *t* test) and reduced blood pressure medications (1 *vs.* 4, *p* < 0.0001 by Mann Whitney *U* test) for patients who underwent surgery compared to those that did not.

## 4. Discussion and Conclusion

PA is the most common cause of secondary hypertension. PA is often a result of APA and PAH, potentially curable causes of PA; surgery can normalize hypokalemia in nearly 100% patients and correct hypertension in 30%–60% of patients [21,22]. We found significant improvement in patients’ blood pressure, with nearly a third of surgically treated patients no longer requiring antihypertensive medications, and a concomitant normalization of potassium levels.

The PA treatment algorithm primarily involves distinguishing unilateral from bilateral disease, as surgery is so effective in treating unilateral causes [20]. While some have suggested that confirmatory laboratory testing may not be necessary in the diagnostic algorithm [23], AVS remains a critical part of the evaluation of patients with PA, as cross-sectional imaging is not always reliable. At least 25%–30% of adrenal adenomas are not detected on abdominal CT [24]. Furthermore, incidental adrenal nodules are discovered on high resolution CT in 4.4% of people [25], and on autopsy in as many as 9% of normotensive and 12% of hypertensive patients [26]. Not only may an adrenal mass not be detected, but if one is discovered, it is likely nonfunctioning.

In this study, we found pathologic concordance for diagnostic lateralizing AVS of 94%. In 22% of patients, diagnostic AVS led to management contradictory to that suggested by pre-procedure CT. In one study of over 200 patients, adrenal CT or MRI was concordant with surgical pathology in only 58.6% of cases, though performed better in patients under 35 years of age [27]. In a systematic review from 2009, CT and MRI were accurate in only 62% of 950 patients [7]. However, more recent studies have shown sensitivity and specificity on the order of 80% [28]. The findings in our study reaffirm the superiority of AVS in accurately diagnosing unilateral aldosterone-producing lesions, and confirms that AVS remains the gold standard in diagnosis.

In our experience, CT and AVS are assessed in conjunction, with each modality providing complementary information. In cases where findings are contradictory, AVS may be repeated for further characterization. Furthermore, when AVS is technically unsuccessful in one adrenal gland, CT findings in conjunction with compelling clinical data can be used to interpret measurements from the contralateral gland, or to guide the decision to repeat AVS or continue with surgery despite nondiagnostic AVS. The management of the 13 patients with technically unsuccessful AVS who nonetheless went on to undergo adrenalectomy demonstrates how cross-sectional, laboratory, and AVS findings may be interpreted together to guide management. All of these patients had adenomas resected, and had significant improvement in blood pressure and potassium levels. These findings are in line with previous studies suggesting that technical failure in assessing one adrenal vein during AVS may not necessarily lead to a clinical failure [29].

Furthermore, patients who were eventually medically managed due to absence of lateralization on AVS had less severe hypertension and hypokalemia compared with the patients with unilateral lesions who were surgically managed. These findings are similar to those in previous reports [30] and suggest that clinical and laboratory evaluation can further help distinguish patients who are surgical candidates in cases where AVS is technically unsuccessful.

The relatively low technical success rate of 63% is multifactorial and represents the experience of one institution over a period of decades. As experience accumulated, technology improved, and performance of the procedure was restricted to fewer operators, technical success improved. These findings are in line with recommendations to consolidate experience in referral centers with greater numbers of cases [20].

The role of contralateral gland suppression as additional data from AVS was not evaluated in our study. Among patients with diagnostic lateralizing AVS who underwent surgical resection, all experienced resolution of hypokalemia, and there was a substantial improvement in hypertension. There were two patients with cortical hyperplasia without a dominant adrenal adenoma on pathology, and were therefore considered as discordant with AVS results. These patients had significant contralateral suppression and significant clinical improvement after surgery. There were no patients with lateralization indices <4 in this study who were considered for surgery; therefore our results do not inform previous suggestions of the important of contralateral suppression in this patient population [31].

The primary limitations of this study are its retrospective nature, entailing loss of some patients to follow up and lack of standardization among referring providers and interventional radiologists performing the procedure. Because most patients were referred from outside institutions, the laboratory evaluation of the patient leading to diagnosis of PA prior to AVS is not available. Furthermore, as thresholds for determining placement of the catheter within the adrenal vein and for determining lateralization are somewhat controversial, interpretation of AVS results may differ between clinicians, leading to variation in management decision-making over the course of the study. Finally, because all adrenal glands are not pathologically sampled or removed, it is not possible to determine sensitivity and specificity, as false negatives are not detected.

In conclusion, AVS is a technically challenging procedure that is critical in the evaluation for surgically curable causes of secondary hypertension. However, even technically unsuccessful sampling procedures offer diagnostic value, when interpreted in the context of clinical, laboratory, and cross-sectional imaging findings.
